# Inferring the Tree of Life: chopping a phylogenomic problem down to size?

**DOI:** 10.1186/1741-7007-9-59

**Published:** 2011-09-20

**Authors:** Olaf RP Bininda-Emonds

**Affiliations:** 1 AG Systematik und Evolutionsbiologie, IBU-Fakultät V, Carl von Ossietzky Universität Oldenburg, Carl von Ossietzky Strasse 9-11, 26111 Oldenburg, Germany

## Abstract

The combination of molecular sequence data and bioinformatics has revolutionized phylogenetic inference over the past decade, vastly increasing the scope of the evolutionary trees that we are able to infer. A recent paper in *BMC Biology *describing a new phylogenomic pipeline to help automate the inference of evolutionary trees from public sequence databases provides another important tool in our efforts to derive the Tree of Life.

See research article: http://www.biomedcentral.com/1741-7007/9/55

## The Tree of Life

The Holy Grail of phylogenetic research is to reconstruct the evolutionary relationships for all of life, currently, if vaguely, estimated to range somewhere between 3 and 100 million species [1]. Hundreds of years of systematic research have arguably yielded a reasonable idea of how the main branches of the Tree of Life are arranged, at least for eukaryotic organisms. Nevertheless, numerous problematic branches naturally remain, as does the question of how the main branches come off the tree trunk. In large part, much of the challenge going forward will be to fill in this scaffold formed by the major branches to provide a complete evolutionary picture of the approximately 1.7 million-and counting-described species on the planet.

In our attempts to derive the Tree of Life, the limiting factor has always been the amount of data available to us. Prior to the molecular revolution, phylogenetic data were comparatively limited, with only morphology being generally available (ignoring early molecular data sources such as DNA-DNA hybridization or immunogenetic and serological data). These data were sufficient to provide us with a general overview, but the resolution was often limited. So, for example, whereas the main groupings, or orders, of eutherian mammals were relatively uncontroversial, their relationships to one another were not. Similarly, the large morphological differences between the animal phyla made them easy to distinguish, but often difficult to place relative to one another.

The growing abundance of DNA sequence data-whether in the form of individual genes, expressed sequence tags (ESTs), or whole genome data-has brought a wealth of new information into play, sometimes contradicting classical hypotheses and often providing more resolution than morphology could alone. The past 15 years have witnessed an explosive growth in sequencing effort and in public databases of sequence information such as GenBank and its sister databases EMBL and DDBJ. Indeed, the amount of information in GenBank is staggering. As of April 2011, the nearly 200 million sequence records in the traditional and whole genome divisions comprised nearly 320 billion bases for almost 250,000 species. The growing use of next-generation and next-next-generation sequencing technologies promises to accelerate the growth rate even further.

**Figure 1:**
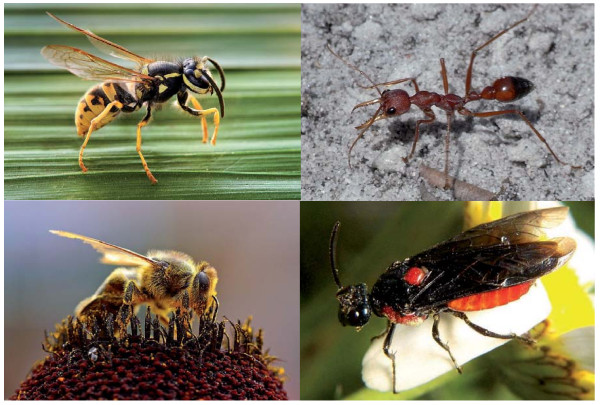
**Representative species of Hymenoptera**. The pictured hymenopteran species clockwise from top left are the German wasp (*Vespula germanica*), red bull ant (*Myrmecia gulosa*), Argid sawfly (*Arge humeralis*) and European honey bee (*Apis mellifera*) (images courtesy of Richard Bartz, user Quartl, Bruce Marlin and Jon Sullivan, respectively; all were obtained from Wikimedia Commons under the Creative Commons Attribution/Share-Alike License except the honey bee, which has been released into the public domain).

**Figure 2:**
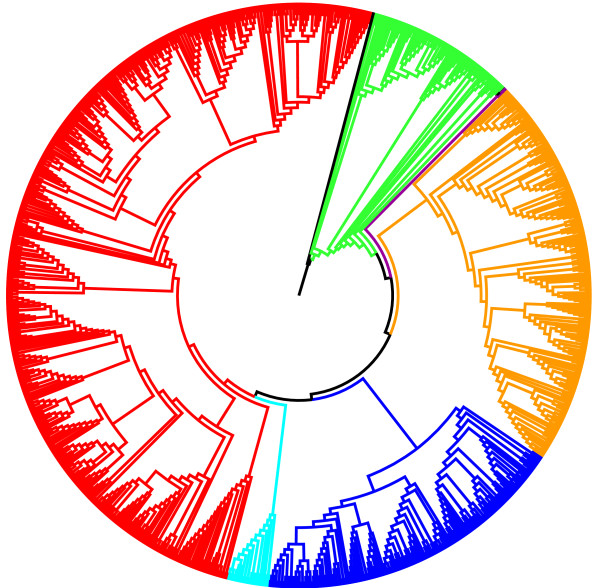
**Phylogenetic tree of the Hymenoptera**. Using a new phylogenomic pipeline that they have developed, Peters *et al*. [3] were able to construct a comprehensive phylogeny across the group (underlying data for the image courtesy of Ralph Peters) from the public sequence data available in GenBank. Key: green, 'Symphyta'; orange, Ichneumonoidea; dark blue, Proctotrupomorpha; cyan, Evanioidea; red, Aculeata.

Despite this, the amount of molecular data remains limited and our data matrices are very sparse, even for well sampled groups such as green plants or mammals [2]. Paradoxically, sparse and limited as the data are, they are still stretching the limits of what we can process currently, from the point of view of both data collection and actual phylogenetic analysis. The phylogenomic pipeline developed by Peters and colleagues [3] represents the latest in a series of automated solutions (for example, [4-7], in addition to those listed in [3]) to both of these problems, all of which are geared to facilitate large-scale phylogenomic analyses using publicly available sequence data. Using their pipeline, they were able to construct a comprehensive molecular tree of over 1,100 species of Hymenoptera (bees, ants, wasps, and sawflies; Figure 1), presenting the state-of-the-art with respect to hypotheses of evolutionary relationships within the group (Figure 2).

Taken together, these phylogenomic pipelines epitomize the revolution that the combination of molecular sequence data and bioinformatics has wrought on phylogenetic research in the past 20 years. Our evolutionary trees are larger and more complete than ever, giving hope that the Tree of Life might be realised soon. However, obstacles still stand in our way.

## Quality control: guarding against the black box

The potential Achilles heel of any automated process is the need to balance minimizing human intervention against ensuring data quality, a trade-off that becomes increasingly relevant as ever larger amounts of data are processed. Phylogenomic pipelines, in particular, must combat the well-known problem that public, non-curated sequence databases like GenBank are replete with errors [8], ranging from simple sequencing errors to more serious problems including contamination, false identifications and erroneous annotations. However, even GenBank's curated RefSeq database is not immune to these problems [9].

The wealth of quality controls (for example, reciprocal BLAST searches against a reference genome) implemented by Peters and colleagues in their pipeline should be sufficient to catch most obvious instances of contamination or widely divergent nuclear copies of mitochondrial genes (or 'numts' as they are commonly known). Because it does not tend to rely on GenBank's gene annotations, the pipeline can also potentially correct for falsely annotated gene sequences. Indeed, such automated orthology assessment (which, it must be pointed out, is not entirely without its limitations) underlies its construction of homologous sets of nuclear genes. The pipeline also builds in cutting edge tools targeting issues known to impact negatively on phylogenetic analysis. Thus, the final data set comprises maximally informative and overlapping alignment subsets that have been pruned of regions of dubious alignment quality across species as well as having been controlled for compositional bias in the sequence data.

Importantly, the Peters *et al*. pipeline, like most others, is only semi-automated, consisting basically of a framework of programs to be used in conjunction with one another. This setup not only permits new tools to be plugged into the pipeline, but also provides the investigator with the opportunity to examine the output at each stage of the process and to catch any errors missed or made by the programs. Whether or not this is done in practice remains to be seen.

Where all phylogenomic pipelines thus far fall short, however, is on the taxonomic side of things. Although GenBank provides a taxonomy of species names compiled using several recognized sources, they readily admit they themselves are not an authoritative taxonomic source. Thus, the sequences for a given species might be split across different taxonomic synonyms. More insidious and worrisome, however, is when sequences have been assigned to the wrong species by the original investigators. Estimates of the frequency of such incidences of taxonomic misidentification are rare, but the few case studies indicate it to be significant, potentially upwards of 20% for some groups [10]. Whereas a component could be built into the pipeline to correct the synonymy problem, tracking down the misidentification problem is difficult at best, and utterly impossible in a black-box approach.

## The computational challenge

Phylogenetic analysis represents a difficult problem computationally because the number of trees increases super-exponentially with the number of species in the analysis. Thus, whereas there are only 3 possible rooted trees that link 3 species, there are already 15 possible trees that link 4 species and 105 that link 5. With only 67 species, we already face a forest of trees (2.8 × 10^111^) to search through that is larger than the volume of the universe in cubic Ångstroms.

In tackling this problem, the input of computer scientists has proven invaluable over the past decade. The combination of more efficient implementations, cleverer heuristic search strategies, and parallel computing means that computationally intensive likelihood-based analyses of a scale that was unthinkable even with methods like maximum parsimony not even a decade ago are now increasingly commonplace. The Peters *et al*. pipeline takes full advantage of these developments, using RAxML, one of the fastest maximum likelihood programs available, for the final phylogenetic analysis. Thus, their analyses of the hymenopteran data sets (over 1,100 species and 80,000 sites) required only 9 days on a medium-size cluster, complete with estimates of support obtained via bootstrapping. (For comparison, parsimony analyses without bootstrapping of the 500 species and 759 sites of the so-called 'Zilla' data set for green plants, one of the largest phylogenetic matrices of its day, required nearly 12 months of CPU time in 1997.) Indeed, the Peters *et al*. matrices only represent medium-size problems for RAxML, which has successfully analysed a data set of nearly 10,000 aligned sites for over 55,000 flowering plant species [11].

But, how accurate are these phylogenomic trees, given that we are searching through the equivalent of an exponential number of universes of forests? Fortunately, the answer would appear to be 'pretty good'. The phylogenies obtained by Peters *et al*. generally reconstruct uncontroversial relationships within Hymenoptera, with unusual groupings being traced back more to issues of data quality (for example, lack of overlap, large amounts of missing data). Similarly, simulation studies show that accuracy is relatively constant (± 90%) across problem sizes ranging from 4 to 4,096 species for numerous methods, including maximum likelihood using RAxML [12]. These represent problem sizes currently being investigated and provide hope that the analysis of even larger problems will be tractable and present the same degree of accuracy in the future.

## Moving forward

The growing ease with which we can generate, collate, and analyse molecular sequence data within a phylogenomic framework has contributed significantly to the dramatic expansion of the scope of systematic research over the past decade. In this, solutions such as the one provided by Peters and colleagues will play an important role in our full-scale assault on the Tree of Life, especially given its open nature and applicability to any taxonomic group of interest. At the same time, however, it is important to remember that these bioinformatic solutions merely represent tools to further our research objectives and cannot replace a critical assessment of both the underlying data and the results they present. The Tree of Life is coming increasingly within our reach, but we still must take care not to grasp automatically at the first solution that comes along.
